# P-1110. Unmasking the transmission of infections in healthcare settings: Cleaning staff- a neglected group in hospital infection control program

**DOI:** 10.1093/ofid/ofaf695.1305

**Published:** 2026-01-11

**Authors:** Md Golam Dostogir Harun, Md Shariful Amin Sumon, Tanjeemay Tamanna, Fairoze Masuda Akther, Antara Swarnali Priyanka, Syed Abul Hassan Md Abdullah, Aninda Rahman, Md Saiful Islam

**Affiliations:** icddrb, Dhaka, Dhaka, Bangladesh; icddr,b, Dhaka, Dhaka, Bangladesh; icddrb, Dhaka, Dhaka, Bangladesh; icddrb, Dhaka, Dhaka, Bangladesh; icddrb, Dhaka, Dhaka, Bangladesh; South Asia Field Epidemiology and Technology Network (SAFETYNET), Bangladesh, Dhaka, Dhaka, Bangladesh; Directorate General of Health Services, Government of Bangladesh., Dhaka, Dhaka, Bangladesh; UNSW, Sydney, New South Wales, Australia

## Abstract

**Background:**

Cleaning staff are essential and integral to infection prevention and control (IPC) in hospital settings, yet their contributions are frequently undervalued in IPC programs. Yet, their role in IPC activities along with their knowledge, attitude and practices towards IPC measures have not been explored in Bangladeshi hospitals. Therefore, this study explored the knowledge-attitude-practice (KAP) along with hand hygiene compliance among cleaning staff in selected hospitals in Bangladesh.

**Figure-1:**
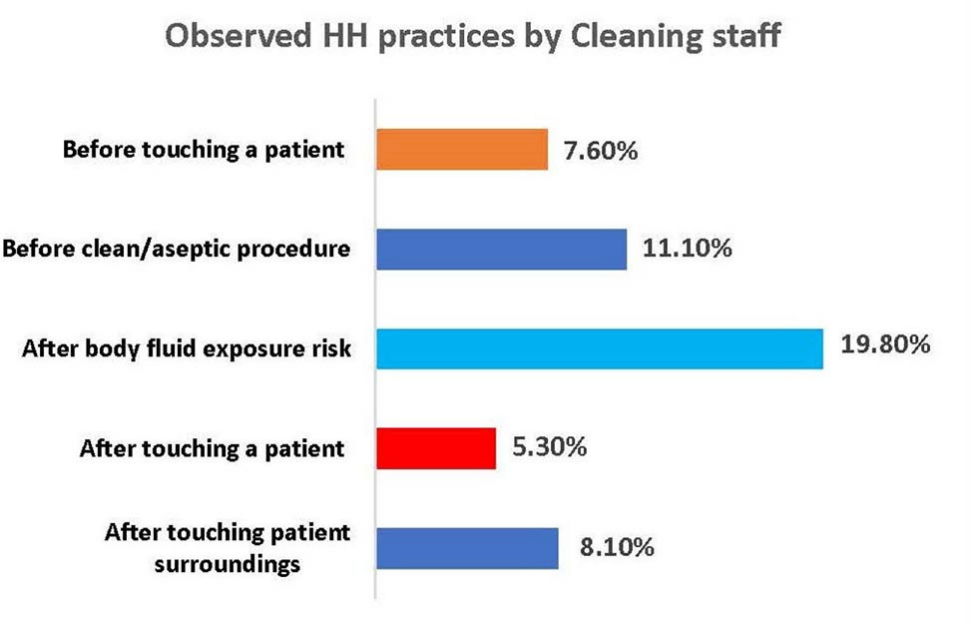
Observed HH practices by Cleaning staff Observed HH practices by cleaning staff

**Figure-2:**
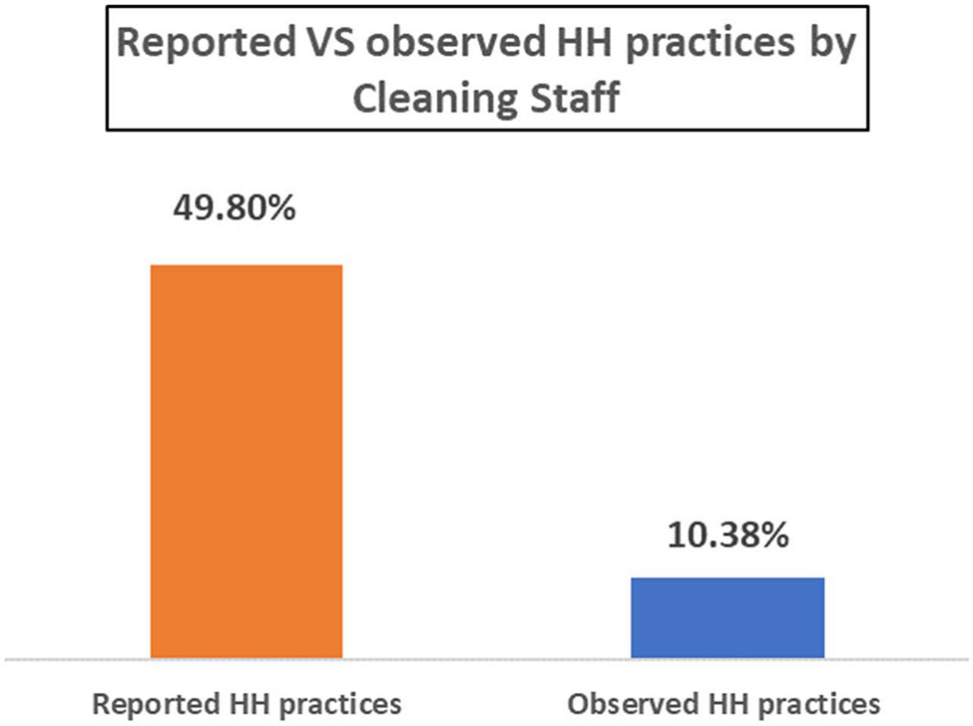
Reported and observed HH practices by cleaning staff Reported and observed HH practices by cleaning staff

**Methods:**

From September to December 2024, we conducted a cross-sectional study and assessed cleaning staff’s KAP related to IPC using a semi-structured questionnaire. KAP components were categorized as “good”, “fair”, or “poor” based on the percentage of positive replies ( >75%, 50-75%, < 50%). In addition, we conducted 8 hours of observation in 48 wards to document hand hygiene (HH) practices against WHO’s 5 moments of HH.

**Results:**

A total of 260 cleaning staff were interviewed, with a mean age of 37.5 years (SD 7.5), with 63.3% possessing an educational attainment below grade 10. Only 13.5% of respondents reported good KAP scores, while 81.9% exhibited fair and rest had poor range of KAP score. The average scores for the KAP categories were 41.9 (SD 13.0), 82.6 (SD 12.5), and 49.8 (SD 16.5), respectively. The observational data indicated a hand hygiene compliance rate of 10.38% (311/ 2,998). According to the 5-moments established by WHO, HH compliance was highest at post-body fluid exposure (19.8%), followed by prior to clean/aseptic procedure (11.1%) and post-touch patient surroundings (8.1%). Lower compliance levels were observed before touching a patient (7.6%), after touching a patient (5.3%), and after touching patient surroundings (8.10%).

**Conclusion:**

This study revealed that cleaning staff’s knowledge and attitudes towards IPC measures did not consistently translate into practice. Therefore, a significant gap remains that may contribute to healthcare-associated infections in hospital settings. Interventions that target engaging cleaning staff in the development of IPC strategies through co-design workshops, regular training, and structured monitoring may improve cleaning staff IPC practices.

**Disclosures:**

All Authors: No reported disclosures

